# Head-Down Tilt Bed Rest Studies as a Terrestrial Analog for Spaceflight Associated Neuro-Ocular Syndrome

**DOI:** 10.3389/fneur.2021.648958

**Published:** 2021-03-26

**Authors:** Joshua Ong, Andrew G. Lee, Heather E. Moss

**Affiliations:** ^1^University of Pittsburgh School of Medicine, Pittsburgh, PA, United States; ^2^Department of Ophthalmology, Houston Methodist Hospital, Houston, TX, United States; ^3^Baylor College of Medicine and the Center for Space Medicine, Houston, TX, United States; ^4^The Houston Methodist Research Institute, Houston Methodist Hospital, Houston, TX, United States; ^5^Departments of Ophthalmology, Neurology, and Neurosurgery, Weill Cornell Medicine, New York, NY, United States; ^6^Department of Ophthalmology, University of Texas Medical Branch, Galveston, TX, United States; ^7^University of Texas MD Anderson Cancer Center, Houston, TX, United States; ^8^Texas A and M College of Medicine, Bryan, TX, United States; ^9^Department of Ophthalmology, The University of Iowa Hospitals and Clinics, Iowa City, IA, United States; ^10^Departments of Ophthalmology, Stanford University, Palo Alto, CA, United States; ^11^Departments of Neurology & Neurosciences, Stanford University, Palo Alto, CA, United States

**Keywords:** spaceflight associated neuro-ocular syndrome, head-down tilt bed rest, astronaut, space medicine, optic disc edema, microgravity, terrestrial analog, countermeasures

## Abstract

Astronauts who undergo prolonged periods of spaceflight may develop a unique constellation of neuro-ocular findings termed Spaceflight Associated Neuro-Ocular Syndrome (SANS). SANS is a disorder that is unique to spaceflight and has no terrestrial equivalent. The prevalence of SANS increases with increasing spaceflight duration and although there have been residual, structural, ocular changes noted, no irreversible or permanent visual loss has occurred after SANS, with the longest spaceflight to date being 14 months. These microgravity-induced findings are being actively investigated by the United States' National Aeronautics Space Administration (NASA) and SANS is a potential obstacle to future longer duration, manned, deep space flight missions. The pathophysiology of SANS remains incompletely understood but continues to be a subject of intense study by NASA and others. The study of SANS is of course partially limited by the small sample size of humans undergoing spaceflight. Therefore, identifying a terrestrial experimental model of SANS is imperative to facilitate its study and for testing of preventative measures and treatments. Head-down tilt bed rest (HDTBR) on Earth has emerged as one promising possibility. In this paper, we review the HDTBR as an analog for SANS pathogenesis; the clinical and imaging overlap between SANS and HDTBR studies; and potential SANS countermeasures that have been or could be tested with HDTBR.

## Introduction

During long-duration spaceflight (LDSF) missions, astronauts undergo a number of microgravity-induced physiological changes such as skeletal muscle atrophy, decreased bone mass, and height change (1–3). The neuro-ocular findings of LDSF include optic nerve head swelling, choroidal folds, cotton wool patches, a hyperopic shift, and retinal nerve fiber layer thickening (3). This constellation of findings was initially termed Visual Impairment and Intracranial Pressure (VIIP) syndrome based on the possibility of increased intracranial pressure (ICP) as the unifying mechanism. Over time however, the role of ICP alone in the condition has evolved and the term Spaceflight Associated Neuro-Ocular Syndrome (SANS) is likely more accurate (3).

SANS has an elevated “Likelihood and Consequence” rating from the NASA Human System Risk Board and accordingly will require mitigation for manned deep space journey and planetary missions of 1–3 years duration (4). In anticipation of these future LDSF exploration missions including flights to Mars, it is imperative to further study SANS, and develop strategies to mitigate it. One potential challenge to studying SANS is that it is uniquely associated with LDSF and the microgravity environment, and therefore is difficult to study on the scale required to understand the disease process. Thus, there is a need for terrestrial analogs (i.e., methods to induce SANS-like findings on earth) to accomplish this. Head-Down Tilt Bed Rest (HDTBR) is one such experimental model that has been used to simulate the effects of microgravity to study multiple physiological systems (5–14), and has become of interest as a terrestrial-based analog for studying SANS and testing potential preventative strategies and interventions, collectively referred to as countermeasures. SANS is characterized by an increased percentage of asymmetric or unilateral disc edema and does not have similar patient demographics found in terrestrial IIH (3, 15).

## Clinical Presentation of SANS

Spaceflight is associated with reduced visual acuity in ~29% of astronauts on short-duration (<6 months) missions and 60% of astronauts after LDSF (≥6 months) due to hyperopic shift (4, 16). In 2011, the first report of hyperopic shift, optic disc edema, cotton wool spots, choroidal folds, globe flattening, and retina nerve fiber layer thickening were reported in seven astronauts following a LDSF (6-month mission) on the International Space Station (ISS) (17). In-flight orbital ultrasound has revealed optic nerve sheath diameter dilation, and post-flight lumbar punctures demonstrated slightly elevated but nearly normal opening cerebrospinal fluid (CSF) pressures in some individuals (4, 18). Intraocular pressure (IOP) increases initially during spaceflight, documented with a 20–25% increase 44 min into spaceflight (19). However, the Lifetime Surveillance of Astronaut Health gathered in-flight IOP data on 15 astronauts who underwent LDSF and found no significant changes between baseline pre-flight IOP, 30th day in-flight IOP, 30 days prior to returning to earth IOP, and post-flight IOP. Thus, suggesting that the acute elevation in IOP during immediate exposure to microgravity trends toward baseline shortly afterwards and stays at baseline throughout the mission (4). Prospective studies of SANS include the utilization of pre- and post-flight brain and orbital magnetic resonance imaging (MRI), retina and optic nerve optical coherence tomography (OCT), orbital ultrasound, cycloplegic refraction, funduscopic examination, and lumbar punctures (LP). Some of these measurements can be performed in-flight but some (e.g., LP, MRI) are logistically not feasible on the ISS (3).

Currently, astronauts utilize corrective plus sphere glasses (in the past “Space Anticipation Glasses”), onboard spaceflight missions as a countermeasure for the microgravity-induced hyperopic shifts (4, 20, 21). Although no permanent vision loss has been reported, choroidal folds and posterior globe flattening may persist years after LDSF (3, 17, 22). Several astronauts have reported refractive changes that have yet to resolve (4).

## SANS Pathophysiology Hypotheses

**Figure 1:**
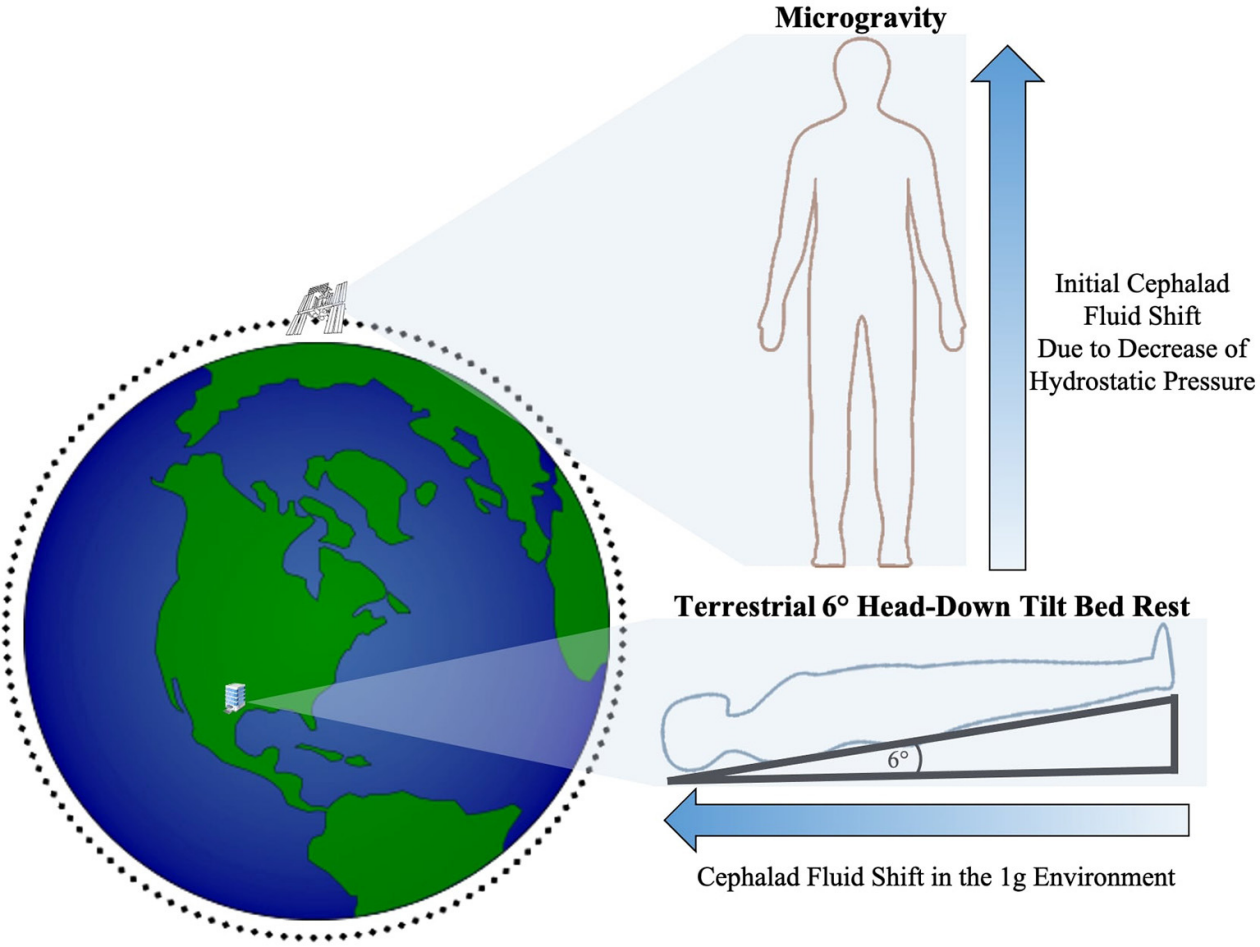
Illustration of the initial cephalad fluid shift in the microgravity environment due to loss of hydrostatic pressure and cephalad fluid shift during terrestrial HDTBR studies (not drawn to scale).

During LDSF, astronauts are exposed to a myriad of factors that impose significant changes to the human mind and body. Microgravity, hypercarbia, and radiation must all be taken into account when understanding the physiologic impact of spaceflight. On Earth, there is a vertical hydrostatic pressure gradient from gravitational downward force resulting in different pressures throughout the body with relative increased pressure in the lower extremities that are closer to the Earth's center (23). A reduction in gravitational acceleration, such as that occurring with travel away from earth, reduces the hydrostatic pressure gradient and allows for a more uniform fluid redistribution in the body with a net shift of fluid toward the cephalad region (3, 23–25) (Figure 1). It is an important distinction that astronauts onboard the ISS are still exposed to ~90% of earth's ground gravitational pull (26). However, they are in a free fall state within the ISS as they orbit at tremendous velocities that counterbalance this gravitational pull, thus inducing the feeling of weightlessness and the physiological effects of microgravity including cephalad fluid migration (3, 23, 25–30).

The exact pathophysiology of SANS continues to be an area of investigation, but multiple hypotheses have emerged. The first theory states that the ocular manifestations arise from high ICP resulting from venous hypertension due to cephalad fluid shifts that occur due to lack of gravity acting on the intravascular fluids. Post-flight MRIs of astronauts have shown findings similar to those seen in terrestrial high ICP states such as idiopathic intracranial hypertension (IIH) including posterior globe flattening and concavity of the pituitary dome (31). Jugular venous distension (JVD) occurs in microgravity studies supporting a hypothesis of venous congestion in the microgravity environment impeding cerebrospinal fluid absorption (3, 32, 33). Furthermore, optic disc edema seen in SANS is a hallmark sign of terrestrial high ICP (3, 34). Lastly, post-flight lumbar puncture readings in astronauts with optic disc edema showed an upper limit of high normal to slightly elevated post flight opening pressures (21–28.5 cm H_2_0), though these may not be representative as they were collected 12–57 days post flight (17). Interestingly, further studies of an astronaut with persistent asymmetric optic disc swelling for 180 days demonstrated only a mildly elevated opening pressure of 22 cm H_2_0 6 days post-flight and normal pressure of 16 cm H_2_0 365 days post-flight (35). In addition, terrestrial IIH symptoms typically include headache and pulsatile tinnitus, whereas the astronauts with optic disc edema do not report these symptoms (36). Interestingly, optic disc edema in astronauts may also persist up to 6 months after spaceflight, long after the proposed cephalic fluid shift resolves whereas the disc edema in IIH patients reduces relatively quickly after reducing pressure in the optic nerve sheath (e.g., Optic nerve sheath fenestration) (35, 36). In addition, terrestrial IIH commonly presents with bilateral optic disc edema in women whereas SANS is characterized by an increased percentage of asymmetric or unilateral disc edema more common in men (3, 4, 35, 36). The collection of these clinical findings eventually led to the conclusion that venous hypertension leading to elevated ICP may not be the sole reason for SANS.

A second hypothesis postulates that in microgravity, CSF pressure increases locally within the optic nerve sheath due to a one-way valve-like mechanism allowing it to enter from the cranial subarachnoid space, but not exit. A similar mechanism of CSF accumulation has been theorized in the past with terrestrial IIH patients who present with persistent optic disc edema despite decreasing ICP (37). CSF biomarker studies have characterized a difference in the CSF composition between the subarachnoid spaces of the optic nerve and around the brain, thus furthering the notion that CSF equilibration between the spaces is incomplete (38). This theory of optic nerve sheath CSF compartmentalization may account for the persistence of optic disc edema in astronauts despite relatively normal to only slightly elevated ICP pressures. In addition, astronauts with SANS exhibit none of the typical terrestrial symptoms of increased ICP (e.g., headaches, diplopia, or tinnitus) (17, 36). Current study of the pathophysiology that induces the compartmentalization of CSF within the optic nerve sheath on Earth may give deeper insight into this theory for SANS (39).

A third hypothesis is that SANS is not due to elevated ICP but rather due to an upward shift of the brain during microgravity (40). Post-flight MRIs in astronauts have shown upward displacement of the optic chiasm (41). This observation is postulated to be due to slight rotation of the brain in microgravity, pulling the optic chiasm upwards and thereby exerting tension on the optic nerve. Because the optic nerve sheath (the dura that surrounds the optic nerve) is connected to the periosteum of the orbital bone, this exerts a compressive force on the optic nerve sheath, which in turn exerts a compressive force on the posterior aspect of the eye. Together these cause deformation of the eyeball (globe flattening) and expansion of the optic nerve sheath without ICP elevation (40). Supporting this hypothesis is a cohort study of twenty-two astronauts with post-flight MRI scans demonstrating a 0.80 ± 0.74 mm (average ± SD) increase in optic nerve length from globe to chiasm compared to pre-flight scans. This was associated with forward displacement of the optic nerve head, which was related to duration of spaceflight and clinical signs of SANS (42).

While the pathogenesis behind SANS is not yet fully understood, the development of these hypotheses allows for proposal of potential experimental models of SANS on Earth (terrestrial analogs). When high ICP was thought to be the main cause, IIH was thought to be a close terrestrial analog but many authors believe that IIH is a flawed model for SANS. However, further characterization of SANS has now led to consideration of other terrestrial analogs that may aid in understanding this neuro-ophthalmic phenomenon (3).

## Head-Down Tilt Bed Rest Studies and Clinical Findings

Terrestrial-based analogs for LDSF have been of longstanding interest in space medicine research as simulated microgravity is an efficient platform to observe physiology in extreme situations. Analogs such as supine bed rest, head-down tilt bed rest (HDTBR), wet immersion, dry immersion, and lower-extremity limb suspension have been previously applied as earthbound experimental models to further understand the human body during spaceflight (24).

HDTBR studies serve as a terrestrial analog by altering the vector of gravitational force on the body to generate a cephalad fluid shift similar to that seen in microgravity (43–45). Subjects lay supine on a bed that has been tilted to lower the head at a specific angle with the international standard angle established at 6° (46). Though other techniques such as parabolic flight more closely simulate the actual microgravity condition, the brief duration of exposure is not sufficient for manifestation of SANS-like features. In contrast, long-duration HDTBR is feasible with study durations of 370 days having been successfully completed (24, 47). Furthermore, many outcomes analogous to those used by NASA for study of astronauts (MRI, OCT, LP etc.) can be measured in the HDT condition (Table 1).

**Table 1:** Spaceflight associated neuro-ocular syndrome (SANS) findings demonstrated in head-down tilt bedrest studies.

**Spaceflight associated neuro-ocular syndrome (SANS) findings**	**Collective head-down tilt findings (up to 70 days)**	**Parameters (angle and duration)**	**References**
Optic Nerve Sheath Distension	**+**	6°, 60 min	(48)
Retinal Nerve Layer Thickening	**+**	6°, 14, 30, & 70 days	(49, 50)
Optic Disc Edema	**+**	6°, 30 days	(50, 51)
Choroidal Thickness Increase	**+**	6°, 60 min	(48, 50)
(Possible) ICP Increase	**+**	10° & 20°, 5 min	(52)
Choroidal Folds	**–**	**–**	–
Hyperopic Shift	**–**	**–**	–
Cotton Wool Spots	**–**	**–**	–

One 6° HDTBR study (14- & 70-days) observed a mild increase in IOP with +1.42 and +1.79 mmHg from baseline, respectively. This study also saw an increase in OCT peripapillary retinal nerve thickening in the 70-day HDTBR compared to the 14-day (+11.50 vs. +4.69 μm, superior peripapillary retinal thickness), though overt optic disc edema was not apparent on clinic exam (49). It was postulated that this RNFL thickening represented early congestion of the optic nerve. Based on the observation that subjects with Ommaya reservoirs, an intraventricular catheter device that acts as a CSF conduit from the ventricle to the scalp, had a reduction of ICP from 14 ± 2 to 10 ± 2 mmHg when moving from a supine position without pillow to with pillow, the research team hypothesized that use of pillows and lifting the torso to eat meals may impact the cephalad venous congestion induced by HDTBR and thus the HDTBR protocol was modified with strict requirements prohibiting any lifting of the head and torso, including raising elbows for support to eat meals, or use of a standard pillow. The protocol was also modified to more closely mimic ISS conditions with a mild hypercapnic environment (PCO_2_ 3.8 mmHg, 0.5%) compared to earth's surface (PCO_2_ 0.6 mmHg, 0.04%), which was verified to not impact arterial PCO_2_ levels (51, 53). A 6°, 30-day HDTBR study using this modified protocol was associated with Frisén grade 1–2 optic disc edema observed in ~45% of subjects (51). This provided strong support for strict HDTBR studies in a hypercapnic environment as an experimental model to study the pathophysiology and test countermeasures for SANS. The same research group also compared healthy subjects undergoing strict HDTBR with 20 astronauts during ~30 days in spaceflight (50). In this study they found a larger increase in peripapillary total retinal thickness in the strict HDTBR subjects than in the 20 astronauts with the average difference being 37 μm. Interestingly, choroid thickness showed the opposite pattern; there was a larger increase in astronauts compared to the strict HDTBR subjects with the average difference being 27 μm. Another study furthered this investigation of the mild hypercapnic environment with HDBTR and observed that individuals who developed SANS features from HDTBR demonstrated elevated reliance on visuals cues when tested on cognitive performance compared to non-SANS HDTBR individuals (54). The study raised concerns that SANS in astronauts may influence inflight performance for certain tasks or that SANS may be associated with cognitive changes. However, a recent strict HDTBR study in the mild hypercapnic environment of approximately 4 mmHg PCO_2_ found no significant change in cerebrovascular reactivity, hypercapnic ventilatory response, or arterialized PCO_2_ (55). Interestingly, observations of cerebral perfusion throughout HDTBR demonstrated decrease in perfusion in all subjects with higher perfusion in the subjects that developed SANS symptoms compared to those who did not (56). With these recent findings, the role of the hypercapnic environment in SANS development remains an area for further study.

HDTBR studies have also been associated with short-term ocular findings that may have implications for astronaut neuro-ophthalmic health. A 7°, 2 min head-down tilt was associated with a decrease in choroidal pulsatile ocular blood flow, suggesting retinal hypoperfusion (57). A 6°, 12-h study HDTBR observed no significant global minimum rim width thinning compared to significant thinning seen in the seated position for 12 h, although it was noted that Bruch's membrane opening height moved anteriorly during HDTBR. The study suggested that this attenuation in neuroretinal rim thinning in postural differences from seated to HDTBR may be due to translaminar pressure difference, which has been proposed as a contributing factor in glaucoma (58). A postural study on ambulatory neurosurgical patients with continuous ICP monitoring observed an increase in ICP during postural change from standing or supine to 10° & 20° head-down tilt (4, 59). 6°, 60-min head-down tilt was associated with optic nerve sheath distension on orbital ultrasound, similar to that seen in astronauts after 1 month in-flight (4, 18, 48), as well as increased subfoveal choroidal thickness on OCT (48). Certain SANS findings such as refractive shift, cotton wool spots, and choroidal folds have not yet been observed in individuals in HDTBR studies.

Some studies have identified possible risk factors for SANS. 15°, 21-min HDT was associated with higher peak IOP in moderate myopes (19.8 mmHg) compared to emmetropes and low myopes (18.6 & 18.7 mmHg, respectively) (60) (Table 2). In another study, single-nucleotide polymorphisms involved in vitamin B_9_ and B_12_ metabolism (5-methyltetrahydrofolate-homocysteine methyltransferase reductase (MTRR) 66 G and serine hydroxymethyltransferase 1 (SHMT1) 1420C alleles) were associated with the magnitude of optic disc edema during strict HDTBR (61). These studies highlight the potential utility of HDTBR to generate hypotheses regarding SANS etiology, identify screening tests, as well as a possible role to empirically screen astronaut candidates.

**Table 2:** Intraocular pressure findings during spaceflight and head-down tilt bedrest studies.

**IOP during spaceflight findings**	**Head-down tilt findings**	**Parameters (angle and duration)**	**References**
Acute IOP Increase	**+**	15°, 21 min	(60)
Subsequent IOP normalization to baseline	Continued IOP increase from baseline	6°, 14 & 70 days	(49)

## Countermeasure Testing and Monitoring Development in Head-Down Tilt Bed Rest

While advancing our understanding of SANS is an important application of HDTBR, the study model is also useful for testing possible countermeasures which may mitigate SANS. Lower body negative pressure (LBNP) device is a non-invasive technique that surrounds the pelvic area and legs and simulates the effects of gravity by inducing fluid redistribution from the cephalad region toward the lower body (52). LBNP has been studied during spaceflight as a countermeasure for cardiovascular responses toward microgravity-induced fluid shifts and orthostatic intolerance upon returning to earth's gravity (62). LBNP of −20 mmHg during a 5-h HDTBR was associated with less increase in optic nerve sheath diameter as well as orbital and intracranial cerebrospinal fluid volume measured with MRI compared to the control group (63). Another HDTBR countermeasure study found a 40% decrease in increased choroid volume in association with use of −20 mmHg LBNP for 8 h/day during 3 days of strict 6° HDTBR. Although the choroid volume was still increased during this HDTBR study, this significant attenuation suggests that LBNP may be an effective countermeasure for SANS (64). Thigh cuffs are also non-invasive devices that have been tested during spaceflight (65). Deflated cuffs are typically placed around the thigh and inflated at specific pressures (often ranging from 40 to 60 mmHg) to limit the amount of fluid flow to the upper body (65). Interestingly, a 15° head-down tilt study utilizing bilateral thigh cuffs at 60 mmHg for 10 min was not associated with significant differences in peripapillary choroidal thickness or optic nerve sheath diameter between cuffed individuals and controls (66).

To mitigate the atrophying effect of microgravity on skeletal muscle, astronauts undergo 2.5 h of intensive resistance and aerobic exercise nearly every day onboard the ISS (67). However, implementation of NASA's integrated resistance and aerobic training (iRAT) protocol during 70-day non-hypercarbic strict 6° HDTBR was not associated with a significant difference in retinal thickening or signs of optic disc edema compared to a control HDTBR group who did not exercise, though IOP was slightly higher (<1 mm Hg) in the exercise group (68). Interestingly, 15° head down tilt for less than an hour was associated with a decrease in IOP in subjects undergoing either moderate-intensity aerobic, resistance, or high-intensity interval aerobic exercise (69). These differences highlight how the impacts of countermeasures are affected by the duration and angle of HDTBR. Integration of results from different HDTBR models is likely necessary to understand the short- and long-term effects.

HDTBR has been leveraged to develop devices for monitoring SANS features that are feasible for inflight use. For example, monitoring ICP during spaceflight is a topic of interest, with invasive measurements such as lumbar puncture currently not feasible inflight. Therefore, deployment of non-invasive devices to measure ICP is desirable (70, 71). Otoacoustic Emission (OAE) phase change is a candidate non-invasive method to monitor ICP. Changes to the positioning and tension of the middle ear caused by ICP changes are detected by a OAE probe placed at the opening of the ear canal. The OAE technique has been tested in head-down tilt and helps guide interpretation of OAE measurements taken aboard the ISS (72). Ocular vestibular evoked myogenic potentials (oVEMPs), which record extraocular muscle activity during vestibular stimulation, were observed to be associated with head-down tilt magnitude, supporting oVEMPs as a non-invasive ICP monitoring tool (73).

For years during spaceflight, OCT has been crucial in quantifying the retinal nerve fiber layer (RNFL) changes to supplement the clinical observations of SANS. In December 2018, OCT angiography (OCTA) became available on the ISS (3). OCTA is a non-invasive, high resolution ocular imaging technique that measures blood flow information and provides angiographic data that corresponds to retinal and choroidal vessels (74). Compared to invasive, contrast enhanced, fundus fluorescein angiography and indocyanine green angiography, OCTA is a non-invasive approach to blood flow visualization in the retina and choroid and may play an increasingly important role in evaluating the ocular vasculature in SANS (3, 75). This technology onboard the ISS will offer a clearer understanding of the volumetric shifts during spaceflight, particularly in the setting of the SANS cephalad fluid theory. Utilizing OCTA with HDTBR studies and comparing these results to ISS OCTA will provide novel information on HDTBR's ability to mimic spaceflight-induced fluid shifts within the retina vasculature.

## Limitations of HDTBR

Although HDTBR is a promising terrestrial analog for investigating SANS and for testing the efficacy of any potential countermeasures, certain limitations must be taken into account. One notable limitation is the small sample size of HDTBR studies, reflecting their time and resource intensive nature. The commitment of subjects to participate for months while maintaining a strict position that may be uncomfortable and induce headaches, which may make recruitment challenging (76). Generalizing HDTBR observations to LDSF and SANS may be limited by differences between terrestrial subjects and astronauts. Two years before a spaceflight mission, astronauts undergo intensive pre-flight strength and aerobic conditioning (77). Matching HDTBR subjects to a similar training regiments prior to HDTBR would be ideal but impractical. Screening protocols, such as a modified Air Force Class III physical exam and clearance by NASA Test Subject Screening facility have been applied to address this and select subjects similar to astronauts in terms of age, height, weight and physical fitness (48).

The variability in conditions for various studies also makes it challenging to integrate the findings of HDTBR with SANS. While a uniform, international standard 6° angle has been established, the strictness of HDT positioning is not standardized and reports of studies often do not include sufficient detail to permit replication. There are conditions induced by HDTBR that are inconsistent with spaceflight, such as subjects having their posterior side in contact with the bed at all times. It is not known how these features impact results and the homology between HDTBR and SANS.

## Discussion and Future Directions

A planned manned mission to Mars will require even longer duration of spaceflight than the longest ISS flights to date. Understanding the pathogenesis; identifying those at increased risk; and mitigating the effects of SANS are of critical importance so that astronauts can complete LDSF missions safely. Terrestrial analogs, that is, experimental models of SANS that can be completed on Earth, are an important tool with which to achieve this and HDTBR is a promising one as, with the right parameters, it is associated with many of the ophthalmic findings seen in SANS. Further HDTBR studies may provide useful information by applying advances in ophthalmic and neuro imaging and image analysis.

HDTBR results support genetic traits such as MTRR and SHMT1 as risk factors. HDTBR itself may be a practical way to screen for risk of SANS development in individuals. HDTBR results also support the use of lower body negative pressure as a countermeasure for SANS. Other countermeasures may include artificial gravity, dietary supplementation, varying training regiments, topical or oral medication, non-invasive monitoring, and external device utilization during or before flight/HDTBR. HDTBR may be helpful to identify the best in-flight measurements with which to diagnose and monitor SANS.

Beyond government-funded space exploration, private space companies (e.g., SpaceX, Blue Origin) strive to increase the accessibility to spaceflight for civilian populations as space tourism (78–80). Short-term HDTBR may be a practical way to screen civilians for susceptibility to consequences of cephalad shifts. Furthermore, a collaborative effort between private space companies and HDTBR researchers may provide novel understandings to short-term spaceflight in the civilian population when comparing pre/post-flight and HDTBR data. As we head into a new era of spaceflight, HDTBR emerges as a promising terrestrial analog to understand how to optimally protect the neuro-ophthalmic health of civilians and astronauts.

## Author Contributions

JO performed the primary literature review and drafted the initial manuscript, tables, and figure, and edited the manuscript. HM and AL provided feedback and edited the manuscript. All authors contributed to the article and approved the submitted version.

## Conflict of Interest

The authors declare that the research was conducted in the absence of any commercial or financial relationships that could be construed as a potential conflict of interest. The Handling Editor declared a past co-authorship with two of the authors, AL and HM.
